# Association of Donor and Recipient Duffy and Kidd Genotypes with GVHD in Leukemia Patients Undergoing Bone Marrow Transplantation

**DOI:** 10.30699/ijp.2025.2061229.3461

**Published:** 2025-11-11

**Authors:** Hawar Nasr Mohammad, Arman Ahmadi, Mehrdad Payandeh, Mahsa Dabir, Mahdi Taqadosi, Fakhredin Saba

**Affiliations:** 1 Department of Medical Laboratory Science, School of Paramedical, Kermanshah University of Medical Sciences, Kermanshah, Iran; 2 Department of Hematology and Medical Oncology, Kermanshah University of Medical Sciences, Kermanshah, Iran; 3 Department of Immunology, School of Medicine, Kermanshah University of Medical Sciences, Kermanshah, Iran

**Keywords:** Graft-versus-host disease (GVHD), Bone marrow transplant (BMT), Kidd blood group, Duffy blood group, Leukemia

## Abstract

**Background & Objective:**

Graft-versus-host disease (GVHD) is a major complication following allogeneic bone marrow transplant (BMT), often limiting therapeutic success in leukemia patients. Chemokine receptors, such as those encoded by Duffy (FY) and Kidd (JK) blood group genes, may influence GVHD development by modulating immune cell trafficking. To evaluate the association between donor and recipient Duffy and Kidd genotypes and GVHD incidence in leukemia patients undergoing HLA-identical sibling BMT.

**Methods:**

This retrospective cross-sectional study analyzed 100 DNA samples from 50 donor-recipient pairs (20 with GVHD, 30 without). Genotyping for FY and JK antigens was conducted using PCR-RFLP. Statistical analysis was performed using chi-square and logistic regression tests in SPSS v19, with significance set at P < 0.05.

**Results:**

Kidd and Duffy genotype distributions differed between BMT recipients who developed GVHD and those who did not. However, when gender was included as an additional variable, these associations in recipients were no longer statistically significant for either genotype. In donors, neither the Kidd nor the Duffy genotypes showed a significant association with GVHD status overall. Interestingly, when stratified by gender, a significant difference was observed only for the Kidd genotype in donors of GVHD-positive recipients, but not in donors of GVHD-negative recipients. However, multivariate logistic regression did not confirm any independent association between Kidd or Duffy genotypes and GVHD in recipients (OR = 2.94, 95% CI: 0.494–17.49, P = 0.236) or donors (OR = 2.273, P = 0.323).

**Conclusion:**

Kidd and Duffy blood group phenotypes may influence susceptibility to GVHD. Understanding this relationship can support better donor-recipient matching in BMT.

## Introduction

Leukemia is a malignancy of the blood-forming tissues, including the bone marrow and the lymphatic system, and is caused by the rapid production of abnormal white blood cells. It accounts for approximately 2.5% of all newly diagnosed cancers and 3.1% of cancer-related mortality worldwide (1). The four major subtypes commonly encountered in clinical practice are acute lymphoid leukemia (ALL), acute myeloid leukemia (AML), chronic lymphoid leukemia (CLL), and chronic myeloid leukemia (CML) (2).

One of the standard treatment methods for leukemia is allogeneic bone marrow transplantation (BMT)**. **BMT, combined with conventional chemoradiotherapy, serves as a complementary strategy in the multidisciplinary treatment of hematologic malignancies (3). Although allogeneic BMT significantly improves survival outcomes, its success is often limited by the occurrence of graft-versus-host disease (GVHD), a serious immunological complication resulting from donor immune cells attacking recipient tissues. Previous analyses suggest that while stem cell source does not significantly impact overall survival, GVHD remains a key determinant of post-transplant outcomes (4).

GVHD is a serious immunological response in which donor-derived immune cells attack the host tissues (5). Chemokines and their receptors play a pivotal role in the pathogenesis of GVHD by guiding the migration of immune cells to target organs (6). Therefore, molecules involved in chemokine transport and response, such as blood group antigens, may influence GVHD development (7).

The Kidd blood group system, identified in 1951 through a case of erythroblastosis fetalis, comprises two codominant alleles: Jka and Jkb (8). These antigens result from a single nucleotide polymorphism in the *SLC14A1* gene, which encodes a urea transporter located on red blood cells. The Jka and Jkb antigens differ by a single amino acid substitution caused by a base change at position 838 in the gene (9). Individuals with the rare null phenotype Jk(a−b−) lack urea transporter activity, rendering their red cells resistant to lysis in 2M urea, a feature widely used to identify this genotype in transfusion practice (10).

The Duffy blood group, first described in 1950, includes the Fya and Fyb antigens. These antigens are encoded by the ACKR1 gene and are expressed not only on red blood cells but also on various non-hematopoietic tissues including vascular endothelium, renal tubular cells, alveolar epithelium, and Purkinje cells of the brain (11-14). The Duffy glycoprotein functions as a non-signaling chemokine receptor known as DARC (Duffy Antigen Receptor for Chemokines), capable of binding several pro-inflammatory chemokines such as IL-8, MCP-1, RANTES, and MGSA (15-17). Through this interaction, DARC is believed to regulate systemic chemokine levels and modulate leukocyte trafficking during immune responses (18, 19). Although its full biological function remains unclear, DARC is implicated in inflammation, tumor progression, and disease susceptibility (20).

Chemokine receptors, including DARC, are thought to play a significant role in GVHD pathogenesis by facilitating leukocyte migration to target organs (21). Recent hypotheses suggest that reduced or absent DARC expression on red blood cells may affect chemokine clearance and transcytosis, potentially altering GVHD risk (13). However, despite these theoretical associations, there is a lack of clinical data evaluating the contribution of Duffy and Kidd genotypes to GVHD occurrence in patients undergoing HLA-identical sibling BMT (22).

Given the immunological importance of these blood group systems and the existing research gap, this study aimed to evaluate the distribution of Duffy and Kidd genotypes in both donors and leukemia patients, and to explore their potential association with the incidence of GVHD following BMT. Understanding these relationships may improve donor selection, support individualized transplant protocols, and contribute to better post-transplant outcomes.

## Materials and Methods

### Study Population and Clinical Management:

This retrospective cross-sectional study was approved by the Ethics Committee on Human Research at Kermanshah University of Medical Sciences (ethical code: IR.KUMS.REC.1403.075). All included patients had a confirmed diagnosis of acute myeloid leukemia (AML) and underwent allogeneic BMT at our center. To minimize clinical variability, all patients were managed under a standardized clinical protocol. This included uniform conditioning regimens, GVHD prophylaxis, and supportive care, all supervised by the same specialized transplant team. Moreover, demographic characteristics such as gender were comparable across study groups, reducing potential confounding effects. 

### Sample Collection

A total of 100 DNA samples were retrieved from the DNA bank of Bostan Clinic (Kermanshah University of Medical Sciences). These included 20 donor–recipient pairs (n = 40) who developed GVHD after allogeneic BMT, and 30 additional donor–recipient pairs (n = 60) who underwent transplantation but did not develop GVHD, serving as the control group.

### Duffy Genotyping

**Fig. 1:**
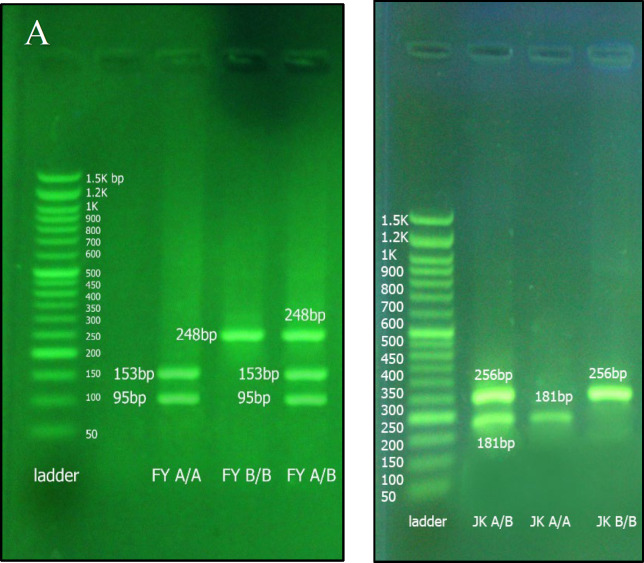
Gel electrophoresis bands of control samples. A) Control Samples of Duffy Genotype:

Genotyping of the Duffy blood group system was performed using the following primers: forward [5′-CTCCCCCTCAACTGAGAACTCAAG-3′] and reverse [5′-AGAGCTGCCAGCGGAAGAG-3′]. PCR was carried out under the following thermal conditions: an initial denaturation at 95°C for 10 minutes, followed by 30 cycles of 30 seconds at 95°C, 30 seconds at 60°C, and 1 minute at 72°C, with a final extension at 72°C for 10 minutes. Amplified PCR products were visualized using 1.5% agarose gel electrophoresis stained with DNA Safe Stain (Pishgam Co., Iran). Genotyping was confirmed by digestion with BanI restriction enzyme (Thermo Fisher Scientific, USA), followed by incubation at 37°C for 16 hours and subsequent electrophoresis. The expected digestion patterns were as follows: FYB homozygous genotype produced a single band of 248 bp; FYA homozygous genotype produced two bands of 153 bp and 95 bp; and the FYA/FYB heterozygous genotype yielded three bands of 248 bp, 153 bp, and 95 bp (Figure 1).

### Kidd Genotyping

Genotyping of the Kidd blood group system was conducted using synthetic oligonucleotide primers: forward [5′-GGCATCTTCTGTGCTCCAGATC-3′] and reverse [5′-CGCCATGAACATTCCTCCC-3′]. PCR was performed under the same cycling conditions as described above. Amplified products were digested with BseLI restriction enzyme (Thermo Fisher Scientific, USA) and incubated at 55°C for 16 hours. Electrophoresis was carried out using 1.5% agarose gel stained with DNA Safe Stain (Pishgam Co., Iran). The expected digestion patterns were as follows: JKB homozygous genotype produced a single band of 256 bp; JKA homozygous genotype produced a single band of 181 bp; and the JKA/JKB heterozygous genotype yielded two bands of 256 bp and 181 bp (Figure 1). All primers were confirmed in silico using NCBI Primer-BLAST. To ensure amplification accuracy, samples with previously confirmed Kidd and Duffy genotypes were included as positive controls in PCR run. PCR products were visualized as single bands of expected size on agarose gel electrophoresis (Figure 1).

### Statistical Analysis

All data were analyzed using IBM SPSS Statistics version 19 (IBM Corp., Armonk, NY, USA). Categorical variables were compared using the Chi-square test (χ²) or Fishe’s exact test where appropriate**.** Continuous variables were analyzed using the independent t-test when assumptions of normality were met. A p-value of less than 0.05 was considered statistically significant. 

Lane 1: DNA size marker; Lane 2: FYA/FYA homozygous genotype (153 and 95 bp fragments); Lane 3: FYB/FYB homozygous genotype (248 bp fragment); Lane 4: FYA/FYB heterozygous genotype (248, 153, and 95 bp fragments).** B) Control Samples of Kidd Genotype**. Lane 1 is DNA ladder base pairs. Lane 2 is JK A/A homozygous genotype fragment (181) bp. Lane 3 is JK B/B homozygous genotype fragment (256) bp. Lane 4 is JK A/B heterozygous genotype fragment (256,181) bp

## Results

In this study, the distribution of Kidd and Duffy blood group genotypes was evaluated in BMT recipients and their respective donors to investigate possible associations with the development of GVHD. The demographic characteristics of the study participants are summarized in Table 1.

### Demographic Variables

No significant associations were found between sex and GVHD status in either recipients (*P = 0.564*) or donors (*P = 0.322*). Likewise, there were no significant differences in age between GVHD-positive and GVHD-negative groups in recipients (*mean ± SD: 37.70 ± 16.21 vs. 39.23 ± 16.10; P = 0.744*) or in donors (*mean ± SD: 37.80 ± 14.11 vs. 36.27 ± 14.33; P = 0.711*) (Table 1).

### Association of Genotype distribution with GVHD incidence

**Figure S2:**
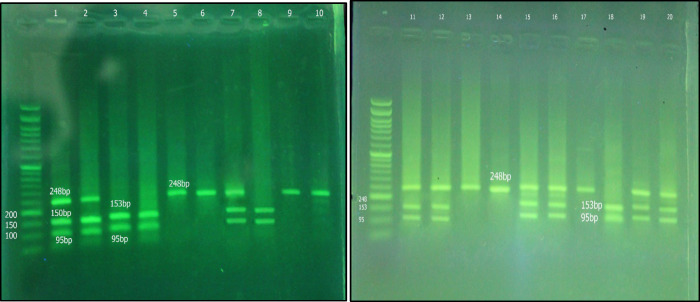
Representative gel electrophoresis showing samples No1-20 of Donors of GVHD-positive recipients- Genotype distribution of Duffy blood group. Samples No. (5, 6, 9, 10, 13, 14, 17) have FY B/B genotype (248 bp); Samples No. (1, 2, 7, 11, 12, 15, 16, 19, 20) have FY A/B genotype (248, 153, 95 bp); Samples No. (3, 4, 8, 18) have FY A/A.

**Figure S3:**
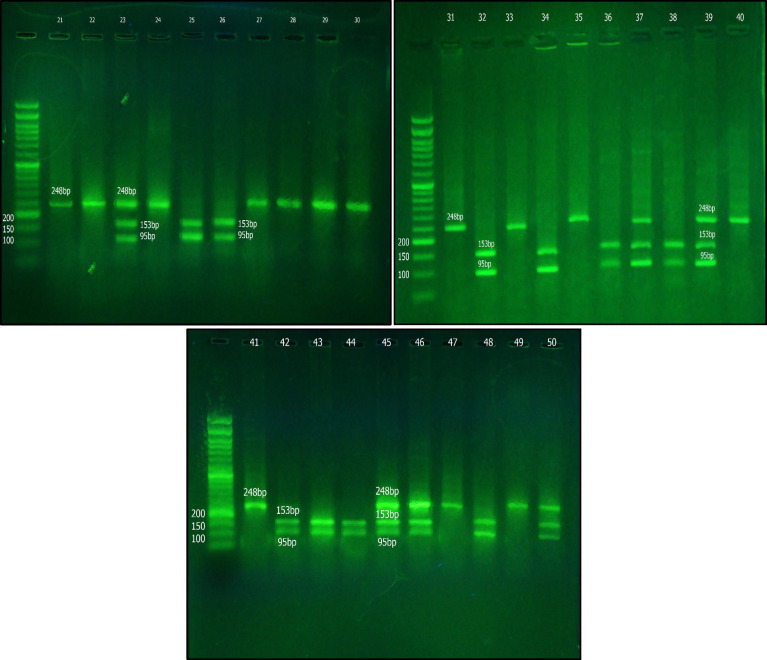
Representative gel electrophoresis showing samples No. 1-20 of GVHD-negative of recipients- Genotype distribution of Duffy blood group. Samples No. (21, 22, 24, 27, 28, 29, 30, 31, 33, 35, 40, 41, 47, 49) have FY B/B genotype (248 bp); Samples No. (23, 37, 39, 45, 46, 50 ) have FY A/B genotype (248, 153, 95 bp); Samples No. (25, 26, 32, 34, 36, 38, 42, 43, 44, 48) have FY A/A.

**Figure S4:**
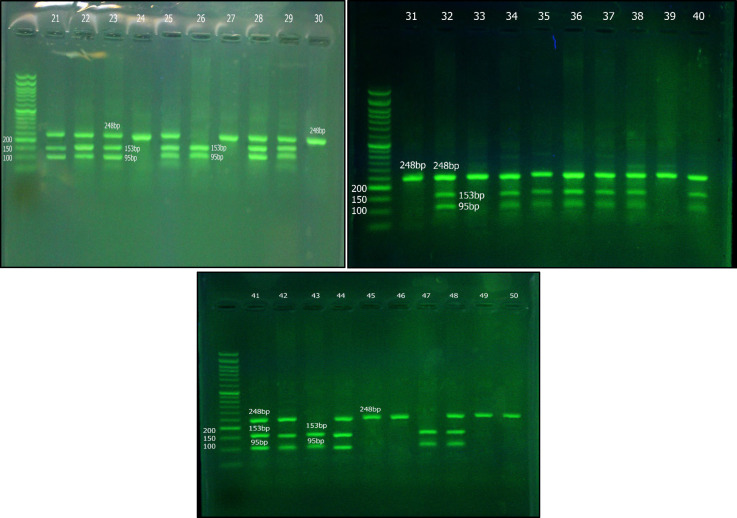
Donors of GVHD-negative recipients - Genotype distribution of Duffy blood group

**Figure S5:**
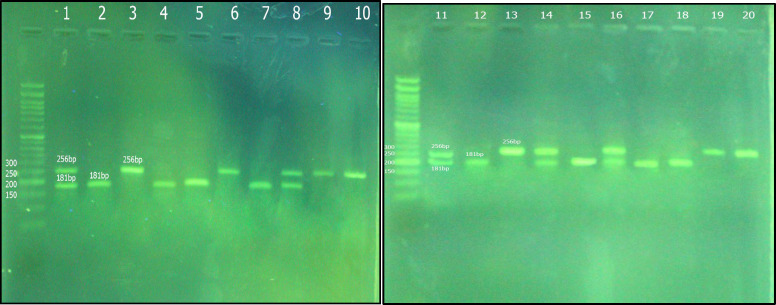
Representative gel electrophoresis showing samples No. 1–20 of GVHD-positive recipients- Genotype distribution of Kidd blood group: Samples No. (2, 4, 5, 7, 12, 15, 17, 18) have JKA/A genotype (181 bp). Samples No. (1, 8, 11, 14, 16) have JK A/B genotype (181,256 bp). Samples No. (3, 6, 9, 10, 13, 19, 20) have JK B/B genotype (256 bp).

**Figure S6:**
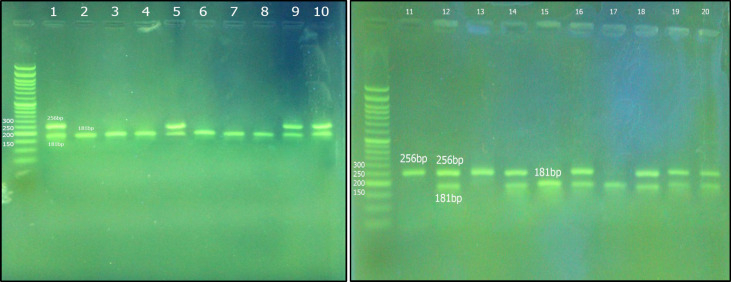
Representative gel electrophoresis showing samples No. 1–20 of Donors of GVHD-positive recipients- Genotype distribution of Kidd blood group: Samples No. (15, 17) have JKA/A genotype (181 bp). Samples No. (1, 5, 9, 10, 12, 14, 16, 18, 19, 20) have JK A/B genotype (181,256 bp). Samples No. (2, 3, 4, 6, 7, 8, 11, 13) have JK B/B genotype (256 bp).

**Figure S7:**
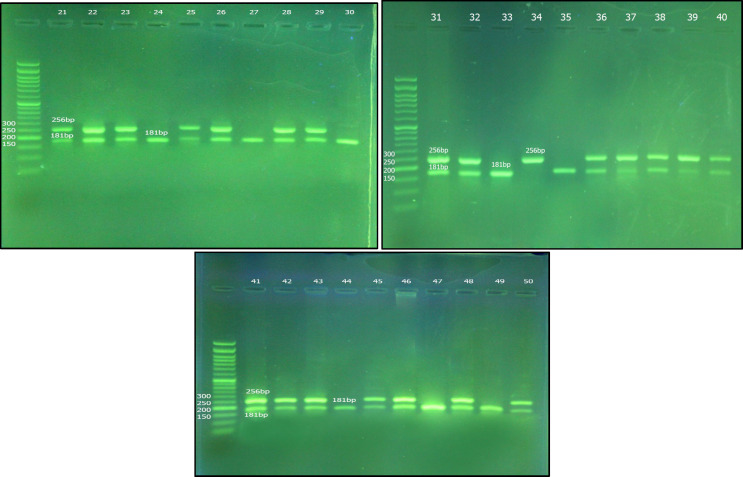
Representative gel electrophoresis showing samples No. 21-50 of GVHD-negative recipients- Genotype distribution of Kidd blood group: Samples No. (24, 27, 30, 28, 33, 35, 44, 47, 49) have JKA/A genotype (181 bp). Samples No. (21, 22, 23, 25, 26, 28, 29, 31, 32, 36, 37, 38, 39, 40, 41, 42, 43, 45, 46, 48, 50) have JK A/B genotype (181,256 bp). Sample No. (34) has JK B/B genotype (256 bp).

**Figure S8:**
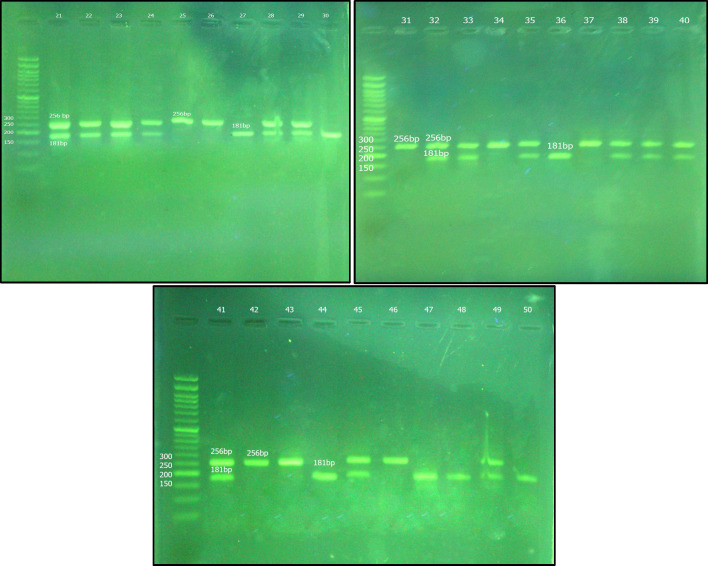
Representative gel electrophoresis showing samples No. 21-50 of Donors of GVHD-negative recipients- Genotype distribution of Kidd blood group: Samples No. (27, 30, 36, 44, 47, 48, 50) have JKA/A genotype (181 bp). Samples No. (21, 22, 23, 24, 28, 29, 32, 33, 35, 38, 39, 40, 41, 45, 49) have JK A/B genotype (181,256 bp). Samples No. (25, 26, 31, 34, 37, 42, 43, 46) have JK B/B genotype (256 bp).

The aim of this part pf the study is to investigate the occurrence of GVHD among patients who have undergone allogeneic BMT, with a specific focus on the distribution of Kidd and Duffy genotypes in both donors and recipients. The analysis was conducted independently of any potential influence of gender on GVHD development, aiming to clarify the isolated contribution of blood group genotype mismatches. Detailed distributions of Kidd and Duffy genotypes are provided in Supplementary Table S1 and Supplementary Figure S2-8.

**Table 1:** Demographic characteristics of BMT recipient and their donors by GVHD status (P: based on independent samples t-test)

Variable	GVHD Positive (n=20)	GVHD Negative (n=30)	*P-Value*
Recipients			
**Total number of recipients**	20	30	
**Male, n (%)**	11 (55%)	14 (46.6%)	0.564
**Female, n (%)**	9 (45%)	16 (53.4%)	
**Age (mean ± SD)**	37.70 (16.21)	39.23±16.10	0.744
Donors			0.322
**Male, n (%)**	12 (60%)	22 (73.3%)	
**Female, n (%)**	8 (40%)	8 (26.7%)	
**Age (mean ± SD)**	37.80 (14.11)	36.27±14.33	0.711
Sibling relationship (Pairs)			
**Brother-Brother pairs, n (%)**	6 (30%)	10 (33%)	
**Sister-Sister pairs, n (%)**	3 (15%)	4 (13.5%)	
**Brother-Sister pairs, n (%)**	5 (25%)	4 (13.5%)	
**Sister-Brother pairs, n (%)**	6 (30)	12 (40%)	

**Table 2:** Comparison of the frequency and percentage of demographic variables and genotype type in recipients and donors (*P: based on chi-square test)

Variable	Categories	Recipients with			Donors of		
		GVHD-positive	GVHD-negative	P	GVHD-positive Recipients	GVHD-negative Recipients	P
Kidd Genotype	JKA/JKA	8 (40%)	8 (26.7%)	*0.001	8 (40%)	7 (23.3%)	*0.282
	JKB/JKB	7 (35%)	1 (3.3%)		2 (10%)	8 (26.7%)	
	JKA/JKB	5 (25%)	21 (70%)		10 (50%)	15 (50%)	
Duffy Genotype	FYA/FYA	1 (5%)	10 (33.3%)	*0.032	4 (20%)	3 (10%)	*0.569
	FYB/FYB	10 (50%)	14 (46.7%)		7 (35%)	10 (33.3%)	
	FYA/FYB	9 (45%)	6 (20%)		9 (45%)	17 (56.7%)	

Note: “Recipients GVHD-positive” indicates patients who developed GVHD after bone marrow transplantation, and “recipients GVHD-negative” indicates patients who did not develop GVHD. “Donors of GVHD-positive recipients” refers to donors whose recipients developed GVHD, while “donors of GVHD-negative recipients” refers to donors whose recipients did not develop GVHD.

### Kidd Genotype

Among allogeneic BMT recipients, a statistically significant difference in Kidd genotype distribution was observed between GVHD-positive and GVHD-negative groups (*P = 0.001*) (Table 2). The JKB**/**JKB genotype was significantly more prevalent in GVHD-positive patients (35%) compared to 3.3% in the GVHD-negative group. In contrast, the JKA**/**JKB heterozygous genotype was more common in GVHD-negative recipients (70%) than in GVHD-positive recipients (25%). The homozygous JKA**/**JKA genotype was relatively evenly distributed (40% vs. 26.7%).

Among donors, although JKB/JKB was more frequent in donors of GVHD-negative recipients (26.7%) than in donors of GVHD-positive recipients (10%), the differences in genotype distribution did not reach statistical significance (*P = 0.282*) (Table 2).

### Duffy Genotype

A significant association was also observed between Duffy genotypes and GVHD in recipients (Table 2) (*P = 0.032*). The FYA/FYA genotype was more frequent among GVHD-negative recipients (33.3%) compared to only 5% in GVHD-positive recipients. Conversely, FYA/FYB was more common in GVHD-positive recipients (45%). Among donors, Duffy genotype distributions were not significantly different between donors of GVHD-positive recipients and donors of GVHD-negative recipients (*P = 0.569*) (Table 2).

### Duffy Genotype Distribution by Sex

Sex-stratified analysis (Table 3) revealed no significant differences in Duffy genotype distribution between males and females in GVHD-positive or GVHD-negative recipients (*P = 0.175* and *P = 0.512*, respectively). Among GVHD-negative donors, the FYA/FYB genotype was more frequent in both sexes, though differences were non-significant.

**Table 3:** Comparison of the frequency of the Duffy genotypes between the two sexes in the recipients and donors of GVHD-positive and GVHD-Negative groups (*P: based on chi-square test or Fisher’s Exact Test)

GVHD Group	Group	Sex	Genotype	n (%)	P-value
Positive	Recipient	Female	FYA/FYA	0 (0.0%)	*0.180
			FYB/FYB	3 (33.3%)	
			FYA/FYB	6 (66.7%)	
		Male	FYA/FYA	1 (9.1%)	
			FYB/FYB	7 (63.6%)	
			FYA/FYB	3 (23.7%)	
	Donor	Female	FYA/FYA	2 (22.2%)	*0.621
			FYB/FYB	4 (44.4%)	
			FYA/FYB	3 (33.3%)	
		Male	FYA/FYA	2 (18.2%)	
			FYB/FYB	3 (27.3%)	
			FYA/FYB	6 (54.5%)	
Negative	Recipient	Female	FYA/FYA	4 (25.0 %)	*0.493
			FYB/FYB	9 (56.3%)	
			FYA/FYB	3 (18.8%)	
		Male	FYA/FYA	6 (42.9%)	
			FYB/FYB	5 (35.7%)	
			FYA/FYB	3 (21.4%)	
	Donor	Female	FYA/FYA	1 (6.3%)	*0.693
			FYB/FYB	5 (31.3%)	
			FYA/FYB	10 (62.5%)	
		Male	FYA/FYA	2 (14.3%)	
			FYB/FYB	5 (35.7%)	
			FYA/FYB	7 (50%)	

**Table 4:** Comparison of the frequency of the Kidd genotypes between the two sexes in the recipients and donors of GVHD-positive and GVHD-negative groups (*P: based on chi-square test or Fisher’s Exact Test).

GVHD Group	Group	Sex	Genotype	n (%)	P-value
Positive	Recipient	Female	JKA/JKA	4 (44.4%)	*0.-980
			JKB/JKB	3 (33.3%)	
			JKA/JKB	3 (22.3%)	
		Male	JKA/JKA	4 (36.4%)	
			JKB/JKB	4 (36.4%)	
			JKA/JKB	2 (27.2%)	
	Donor	Female	JKA/JKA	7 (87.5%)	*0.001
			JKB/JKB	0 (33.3%)	
			JKA/JKB	1 (12.5%)	
		Male	JKA/JKA	1 (8.3%)	
			JKB/JKB	2 (16.7%)	
			JKA/JKB	9 (75%)	
Negative	Recipient	Female	JKA/JKA	3 (18.8%)	*0.304
			JKB/JKB	0 (0.0%)	
			JKA/JKB	13 (81.3%)	
		Male	JKA/JKA	5 35.7%)	
			JKB/JKB	1 (7.1%)	
			JKA/JKB	8 (57.1%)	
	Donor	Female	JKA/JKA	3 (37.5%)	*0.304
			JKB/JKB	3 (37.5%)	
			JKA/JKB	2 (25.1%)	
		Male	JKA/JKA	4 (18.2%)	
			JKB/JKB	5 (22.7%)	
			JKA/JKB	13 (59.1%)	

**Table 5:** Comparison of the frequency distribution of the Duffy genotypes in the recipients and donors of Affected and GVHD-negative groups (*FYA/FYB genotype is the reference category).

Variable	Duffy genotype	Recipients			Donors		
		Odds ratio	95% CI	P	Odds ratio	95% CI	P
FYA/FYA	GVHD-positive	0.485	0.081 to 2.91	0.428	2.625	0.527 to 13.07	0.239
	GVHD-negative	Reference category			Reference category		
FYB/FYB	GVHD-positive	0.742	0.209 to 2.63	0.644	1.5	0.406 to 5.54	0.543
	GVHD-negative	Reference category			Reference category		

### Kidd Genotype Distribution by Sex

Table 4 summarizes the association between the Kidd genotype and GVHD occurrence, stratified by donor and recipient sex. Among recipients, there was no significant relationship between Kidd genotype and GVHD status when comparing male and female patients (P = 0.980 in GVHD-positive, P = 0.304 in GVHD-negative). In contrast, a significant association was found between Kidd genotype and GVHD occurrence among donors of GVHD-positive recipients when comparing male and female donors. However, no significant association was observed between Kidd genotype and the absence of GVHD in donors.

### Duffy Genotype: Multivariate Association (Logistic Regression Analysis)

In logistic regression (Table 5), using FYA/FYB as the reference category, neither FYA/FYA nor FYB/FYB genotypes showed significant associations with GVHD occurrence in **recipients** (*FYA/FYA OR: 0.485, 95% CI: 0.081–2.91, P = 0.428; FYB/FYB OR: 0.742, 95% CI: 0.209–2.63, P = 0.644*). Among **donors**, FYA**/**FYA showed a non-significant trend toward higher GVHD risk (*OR: 2.625, P = 0.239*).

### Kidd Genotype: Multivariate Association (Logistic Regression Analysis)

Similarly, logistic analysis for Kidd genotypes (Table 6), with JKA/JKB as the reference, did not show significant associations with GVHD. In recipients, the JKB/JKB genotype had an OR of 2.94 (95% CI: 0.494–17.49, *P = 0.236*), while in donors, the OR was 2.273 (P = 0.323), both not statistically significant.

**Table 6:** Comparison of the frequency distribution of the Kidd genotypes in the recipients and donors of GVHD-positive GVHD-negative groups (*JKA/JKB genotype is the reference category).

Variable	Kidd genotype	Recipients			Donors		
		Odds ratio	95% CI	P	Odds ratio	95% CI	P
JKA/JKA	GVHD-positive	0.754	0.166 to 3.42	0.714	1.753	0.499 to 6.17	0.381
	GVHD-negative	Reference category			Reference category		
JKB/JKB	GVHD-positive	2.94	0.494 to 17.49	0.236	2.273	0.446 to 11.59	0.323
	GVHD-negative	Reference category			Reference category		

## Discussion

In this study, we investigated the association between recipient JK and FY blood group genotypes and the incidence of GVHD following BMT. Our findings revealed a statistically significant association between the JK genotype and GVHD occurrence in recipients, as well as a significant relationship between JK genotype and GVHD development among donors of GVHD-positive recipients when analysed by gender. These observations suggest that JK antigens may function as minor histocompatibility antigens (mHAgs), eliciting immune responses post-transplantation. This aligns with previous research indicating that mismatches in red blood cell antigens, including Kidd, can contribute to alloimmune reactions in transplant settings. For instance, Rowley et al. discussed the immunohematological consequences of red cell-incompatible transplantation, highlighting that antigens like Kidd could increase the risk of GVHD due to their expression on endothelial and epithelial tissues (23). Interestingly, Kidd antigens are not only limited to red blood cells but are also involved in urea transport in kidney and other epithelial tissues(24). This wider distribution suggests that they might contribute to immune interactions beyond transfusion settings, which could help explain their possible role as minor histocompatibility antigens in BMT (24).

Our study found an initial significant association between FY genotypes and GVHD incidence in recipients; however, this association did not persist when stratified by gender or analyzed in multivariate regression. This finding suggests that the observed relationship may be partly due to confounding factors or the limited statistical power of our sample. This is consistent with the findings of a study conducted on Tunisian patients, which reported no significant correlation between FY antigen disparities and the incidence of either acute or chronic GVHD. The lack of association in both studies may be attributed to the low immunogenicity of FY antigens in the context of BMT (22). The Duffy antigen is also known to act as a receptor for certain chemokines on endothelial cells, which may influence how immune cells move and interact during the immune response after transplantation(25). However, its specific role in GVHD remains to be clarified. It is important to note that our study did not differentiate between acute and chronic forms of GVHD. Given that these two forms have distinct pathophysiological mechanisms and clinical manifestations, future research should aim to stratify GVHD cases accordingly to better understand the specific roles of JK and FY antigens in each subtype.

This study has several strengths that add value to its findings. It is one of the few investigations to simultaneously examine both Kidd and Duffy blood group genotypes in relation to GVHD occurrence following allogeneic HLA-identical sibling transplantation. By focusing specifically on AML patients treated with a standardized conditioning regimen and GVHD prophylaxis, the study minimizes potential confounding from disease heterogeneity and treatment differences. Detailed genotype distributions for both donors and recipients were analyzed, and further stratification by sex provides new insights into the possible role of minor histocompatibility antigens and the influence of demographic factors. All genotyping procedures were carefully validated through in silico primer design and confirmed by RFLP digestion, with representative gel electrophoresis images and complete genotype counts included as supplementary materials. This enhances the accuracy, reproducibility, and methodological transparency of the study. By highlighting the potential modifying effect of sex, the findings emphasize the importance of considering demographic variables in future GVHD risk assessments and donor selection strategies.

Nevertheless, this study has several limitations: its retrospective cross-sectional design restricts causal inference and may involve incomplete data. The relatively small single-center sample, lack of correction for multiple comparisons, and absence of data on factors like CMV status may limit the generalizability and statistical power. Furthermore, selecting participants based on GVHD status could have introduced selection bias. Larger multicenter studies are needed to validate these findings and adjust for potential confounders.

## Conclusion

In summary, our findings indicate that the Kidd and Duffy blood group genotype may play a significant role in the development of GVHD in both recipients and donors undergoing allogeneic BMT. Given the limitations of our single-center study and its relatively small sample size, larger multicenter studies are needed to confirm these preliminary results and clarify the potential utility of Kidd antigen typing in donor selection and GVHD risk assessment.

## Figures and Tables

**Figure S1:**
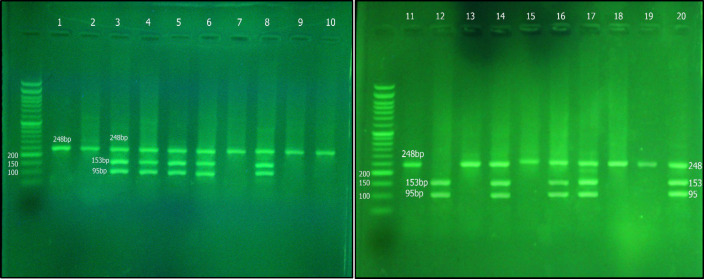
Representative gel electrophoresis showing samples No. 1–20 of GVHD-positive recipients-Genotype distribution of Duffy blood group: Samples No. (1, 2, 7, 9, 10, 11, 13, 15, 18, 19) have FY B/B genotype (248 bp). Samples No. (3, 4, 5, 6, 8, 14, 16, 17, 20) have FY A/B genotype (248, 153, 95 bp). Sample No. 12 has FY A/A genotype.

Samples No. 21 to 30: Samples No. (21, 22, 23, 25, 28, 29) have FY A/B genotype, samples No. (24, 27, 30) have FY B/B genotype, and sample No. 26 has FY A/A genotype. Samples No. 31 to 40: Samples No. (31, 33, 39) have FY B/B genotype, and samples No. (32, 34, 35, 36, 37, 38, 40) have FY A/B genotype. Samples No. 41 to 50: Samples No. (41, 42, 44, 48) have FY A/B genotype, samples No. (43, 47) have FY A/A genotype, and samples No. (45, 46, 49, 50) have FY B/B genotype

## Data Availability

The data supporting the results of this study are available upon request from the corresponding author.
